# α-Mangostin Extraction from the Native Mangosteen (*Garcinia mangostana* L.) and the Binding Mechanisms of α-Mangostin to HSA or TRF

**DOI:** 10.1371/journal.pone.0161566

**Published:** 2016-09-01

**Authors:** Ming Guo, Xiaomeng Wang, Xiaowang Lu, Hongzheng Wang, Peter E. Brodelius

**Affiliations:** 1 School of Science, Zhejiang Agricultural & Forestry University, Lin’an 311300, China; 2 School of Forestry and Bio-technology, Zhejiang Agricultural & Forestry University, Lin’an 311300, China; 3 Department of Chemistry and Biomedical Sciences, Linnaeus University, 391 82 Kalmar, Sweden; University of Hyderabad, INDIA

## Abstract

In order to obtain the biological active compound, α-mangostin, from the traditional native mangosteen (*Garcinia mangostana* L.), an extraction method for industrial application was explored. A high yield of α-mangostin (5.2%) was obtained by extraction from dried mangosteen pericarps with subsequent purification on macroporous resin HPD-400. The chemical structure of α-mangostin was verified mass spectrometry (MS), nuclear magnetic resonance (^1^H NMR and ^13^C NMR), infrared spectroscopy (IR) and UV-Vis spectroscopy. The purity of the obtained α-mangostin was 95.6% as determined by HPLC analysis. The binding of native α-mangostin to human serum albumin (HSA) or transferrin (TRF) was explored by combining spectral experiments with molecular modeling. The results showed that α-mangostin binds to HSA or TRF as static complexes but the binding affinities were different in different systems. The binding constants and thermodynamic parameters were measured by fluorescence spectroscopy and absorbance spectra. The association constant of HSA or TRF binding to α-mangostin is 6.4832×10^5^ L/mol and 1.4652×10^5^ L/mol at 298 K and 7.8619×10^5^ L/mol and 1.1582×10^5^ L/mol at 310 K, respectively. The binding distance, the energy transfer efficiency between α-mangostin and HSA or TRF were also obtained by virtue of the Förster theory of non-radiation energy transfer. The effect of α-mangostin on the HSA or TRF conformation was analyzed by synchronous spectrometry and fluorescence polarization studies. Molecular docking results reveal that the main interaction between α-mangostin and HSA is hydrophobic interactions, while the main interaction between α-mangostin and TRF is hydrogen bonding and Van der Waals forces. These results are consistent with spectral results.

## Introduction

**Fig 1:**
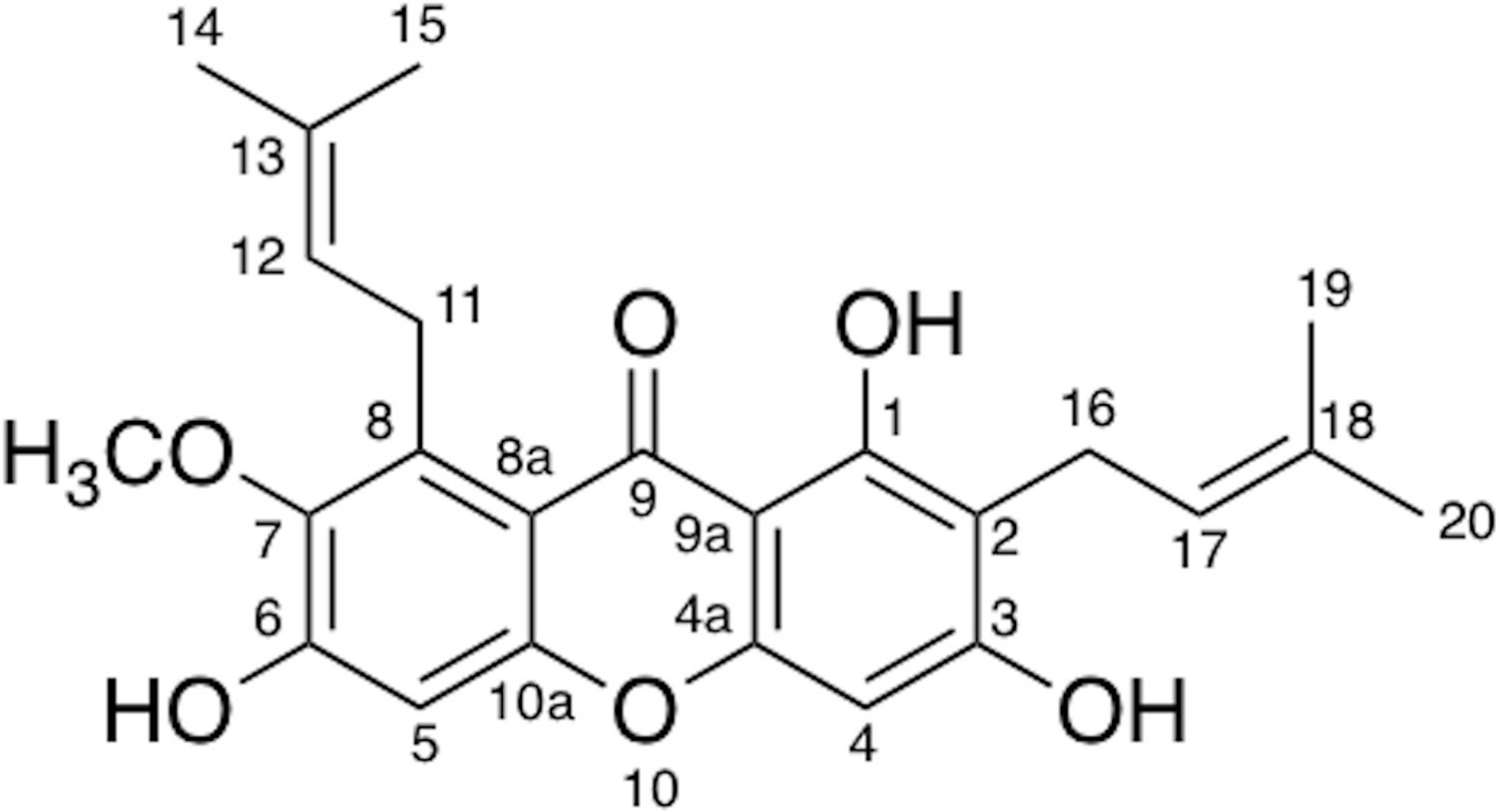
The chemical structure of α-mangostin.

Mangosteen (*Garcinia mangostana* L.) is a member of the *Garcinia* genus, which mainly grows in Thailand, Vietnam, Malaysia, Indonesia, Philippines and other Southeast Asian countries. It is also widely cultivated in Guangxi, Hainan and Zhejiang provinces as well as other areas of China. Its rind has been used as traditional medicines in Southeast Asia to treat abdominal pain, diarrhea, dysentery, cholera, infectious wounds, purulence, chronic ulcers and other diseases. In addition, it also exhibits the effect of anti-inflammatory, antibacterial, anti-malarial, lowering blood pressure, anti-oxidation, anti-HIV, immune regulation, and many other pharmacological activities. It has been established that mangosteen contains a variety of active ingredients, including xanthones, phenolic acids, polysaccharides and pigments [1–6]. Xanthones are the main active substance in mangosteen, wherein α-mangostin (1,3,6-trihydroxy-7-methoxy-2,8-bis(3-methyl-2-butenyl)-9H-xanthen-9-one) (Fig 1) is one of the most important natural xanthone derivative [7]. It can be used as a drug for the treatment of diabetes, reduction of blood lipids, cardiovascular protection, inhibiting leukemia HL-60 cells growth and inhibiting HIV-1 protease. As a health care product, it has the function of antioxidant and anti-aging [8] and it is also used in cosmetics.

α-Mangostin is a natural organic compound with low polarity. Therefore, the common technology of extraction and separation of α-mangostin includes silica gel column separation. However, this method is expensive and it involves a large amount of toxic organic solvents in the separation process. Thus, macroporous resin separation was used to isolate α-mangostin in our study. The cost of macroporous resin is low and the resin is easily regenerated for reuse. The resin can be used repeatedly, and the preparations of extract and separation processes are also simple, low-cost, and suitable for large-scale industrial production. This is the reason why macroporous resin is widely used in separation of natural organic compounds. To the best of our knowledge, the separation of α-mangostin from native mangosteen (Zhejiang province of China) using the macroporous resin method has not been reported before.

Proteins are the main embodiment and implementer of biological functions and studies on their expression pattern and functional model are necessary for the development of life science. Such studies are of great significance for the discovery and screening of pathogenesis, diagnosis and treatment of new drug targets [9–17]. Various proteins can transport fatty acids, bile pigments, amino acids, steroids, metal ions, and many therapeutic molecules in body fluids, while maintaining normal osmotic pressure of blood at the same time. By analyzing the interactions between drugs and transporting proteins under physiological conditions from different points of view, not only can we obtain pharmacodynamic information and elucidate the delivery mechanism of the drug, but we can also provide theoretical references for drug analysis. As one of the important aspect of transporting proteins, serum protein has wide applications in pharmacological research. Approximately 10000 different serum proteins have been predicted, but only around 1500 of these have been identified. Among them, human serum albumin (HSA) and transferrin (TRF) are two important high abundant serum proteins. These two proteins have been crystallized and their three dimensional structures determined. Thus, they are chosen as model protein molecules in this study [18,19].

Human serum albumin (HSA), the most abundant multi-function and multi-purpose protein of plasma, contains 585 amino acid residues, 17 disulfide bonds and its molecular weight is about 67 kD. HSA consists of three structural domains: domain I, domain II and domain III and contains only one tryptophan residue (Trp) locates at position 214. The structure of each domain is divided into two subdomains, A and B, which each is made up of three α-helices [20,21].

Human transferrin (TRF) is the main iron protein in plasma and its function is to transport iron ions from absorption and storage places to the tissue needing iron ion in our body. TRF is a non-heme iron β-globin, containing 679 amino acid residues, 19 disulfide bonds and protected by two N-linked and one O-linked glycosylations. The molecular weight is about 77 kD [22,23].

So far, studies on the interaction of α-mangostin with human serum albumin (HSA) or transferrin (TRF) have not been reported. In our study, α-mangostin was isolated by the HPD-400 macroporous resin method. Combining spectroscopy and molecular modeling methods, the interaction mechanism between α-mangostin and the two serum proteins was investigated. The results also give information on the interaction mechanism between native α-mangostin and HSA or TRF at the molecular level, thereby providing an important reference for the analysis of the pharmacodynamic mechanism of α-mangostin.

## Results and Discussion

### Macroporous resin separation of α-mangostin

The HPD-400 macroporous resin was used to separate α-mangostin in this study. A schematic diagram of the separation system of macroporous resin extraction apparatus is shown in S1 Fig.

The quantity of extracted α-mangostin was measured gravimetrically after each extraction and the yield (%) was calculated as the percent ratio of the mass of extracted α-mangostin to the mass of native mangosteen pericarp powder loaded into the extraction vessel, as in Eq (1).

$$\text{yield}\;(\%)=\frac{\text{mass of extracted}\;\alpha \text{-mangostin}}{\text{mass of mangosteen}}\times 100$$ (1)

**Fig 2:**
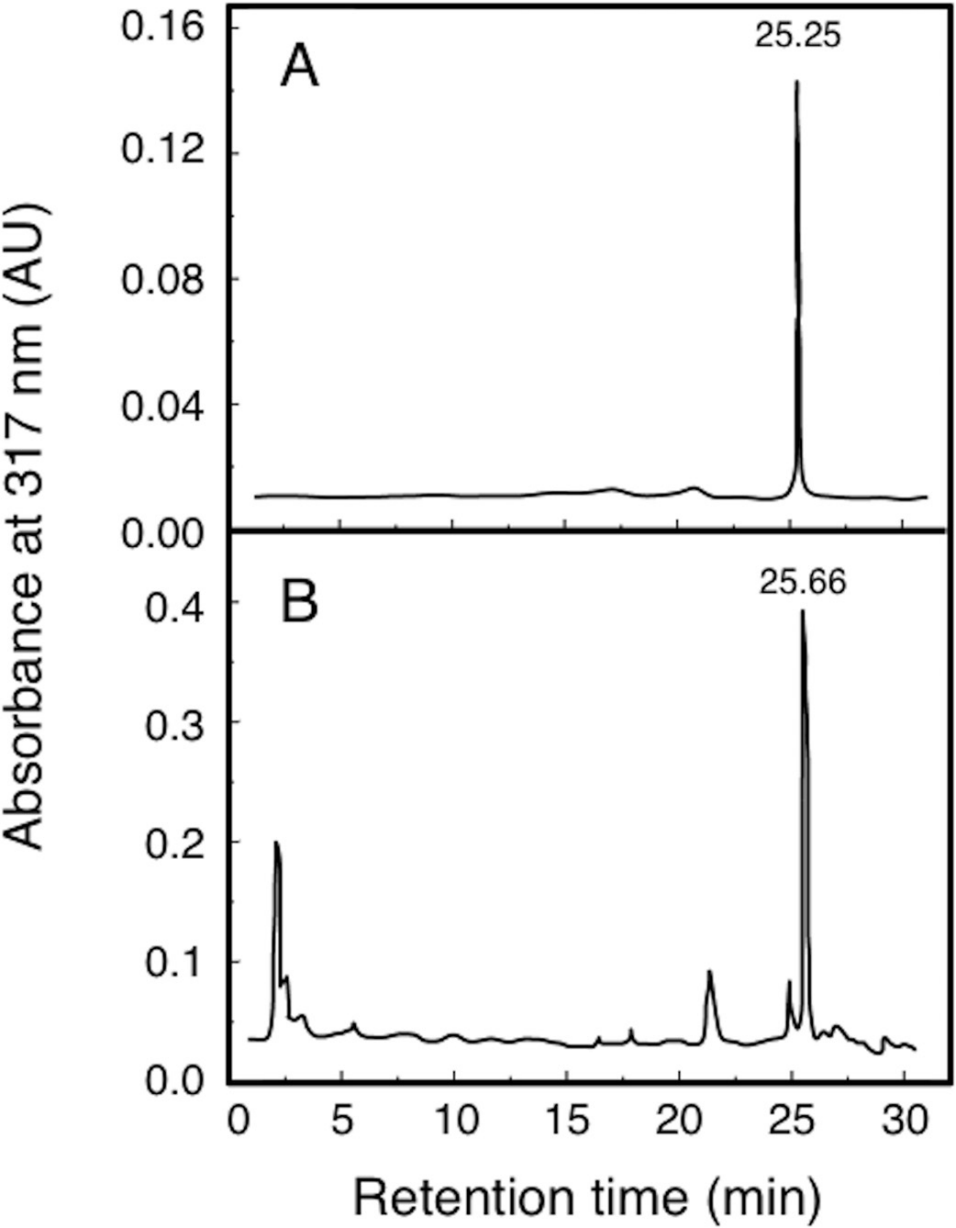
HPLC elution profiles of α-mangostin standard and sample.

High performance liquid chromatography (HPLC) was used to verify the α-mangostin product, as well as, the efficiency of extraction and separation using the HPD-400 macroporous resin. It is shown in Fig 2 that the major peak of the sample has the same retention time as the standard sample, which demonstrated that the samples contained α-mangostin. The relative content of α-mangostin was determined with area normalization method from Fig 2. The overall yield of α-mangostin was 5.2% and the purity as determined by HPLC was 95.6%.

### Structural identification of α-mangostin

α-Mangostin was extracted and separated using HPD-400 macroporous resin. GC-MS and NMR spectroscopy was utilized to identify the recrystallized α-mangostin. The results of mass spectra, ^1^H- NMR (deuterated chloroform), ^13^C - NMR (deuterated chloroform), IR- and UV-spectra are shown as follows: HPLC *R*_*f*_ = 25.25 for α-mangostin standard sample *R*_*f*_ = 25.66 for the purified α-mangostin. ESI-MS (m/z): 409 [M], 411 [M + 2], 355 [M—C_4_H_6_]. UV *λ*_max_: 213, 245, 332. IR: *ν*_max_: 3620, 3420, 3260: [-OH]; 2920, 2860, 2720: [-CH3, -CH2-]; 1640, 1610, 1410, 1380: [Ph]; 1280, 1190: [-O-]; 1080: [-C-O-]; 995: [-CH = CH-]; 850, 667: [Ph-H]. ^1^H-NMR (400 MHz, CDCl_3_):1. 65 (6H, s, 19- and 20-CH_3_), 1.73 (6H, s, 14- and 15-CH_3_), 3.698 [(1H-11, and 2H-16, d)], 3.77 (3H, s, 7-OMe), 5.69 (1H, s, C-3-OH), 5.843 (2H, t, 12- and 17-H), 6.155 (1H, s, H-4), 6.677 (1H, s, H-5), 13.698 (1H, s, C-1-OH). ^13^C-NMR (100 MHz, CDCl_3_): 16.568 (C-15 and C-19), 20.235 (C-16), 24.238 (C-11), 25.064 (C-14 and C-20), 59.307 (7-OMe), 91.383 (C-4), 100.93 (C-9a), 101.601 (C-5), 109.251 (C-2), 109.841 (C-8a), 123.049 (C-11,12), 129.634 (C-13,18), 136.173 (C-8), 142.806 (C-7), 153.932 (C-6), 154.373 (C-10a), 156.085 (C-4a), 159.759 (C-3), 161.663 (C-1), 181.052 (C-9).

The analysis results of the IR- and UV-spectra are summarized in Table 1 and Table 2, respectively.

**Table 1:** The results of IR spectra of α-mangostin.

Wavenumber/cm^-1^	Group
1380	symmetric bending vibration of -CH_3_
2860, 2920	stretching vibration of C-H
1640	stretching vibration of C = C
1460, 1610	skeletal vibration of C = C of aromatic hydrocarbon
850	flexural vibration of C-H of aromatic hydrocarbon
1080, 1190	stretching vibration of C-O connected to hydroxyls
3260, 3420	stretching vibration of intermolecular hydrogen bonding
3620	stretching vibration of free hydroxyls
667	distortion of vibration of O-H
1280	stretching vibration of C-O-C of -OCH_3_ on the benzene ring
1080, 1190	stretching vibration of C-O-C
1280, 1640	stretching vibration of C = O of aromatic ketones
2360	Asymmetric stretching vibration (noise) of CO_2_

**Table 2:** The results of UV spectra of α-mangostin.

Wavenumber/nm	Group
360	*R* area of ketone by *n* → π* transition
213	*K* area contains conjugated system
245	enol type structure

Through the analysis, we confirmed that the crystallized sample is α-mangostin of high purity. The IR, UV, MS and NMR spectral results were in accordance with those previously reported [24,25]. The IR- and UV-spectral results conclusively confirmed the structure of the sample.

### The fluorescence spectra and conjugation reaction mechanism of the interaction between α-mangostin and HSA or TRF

**Fig 3:**
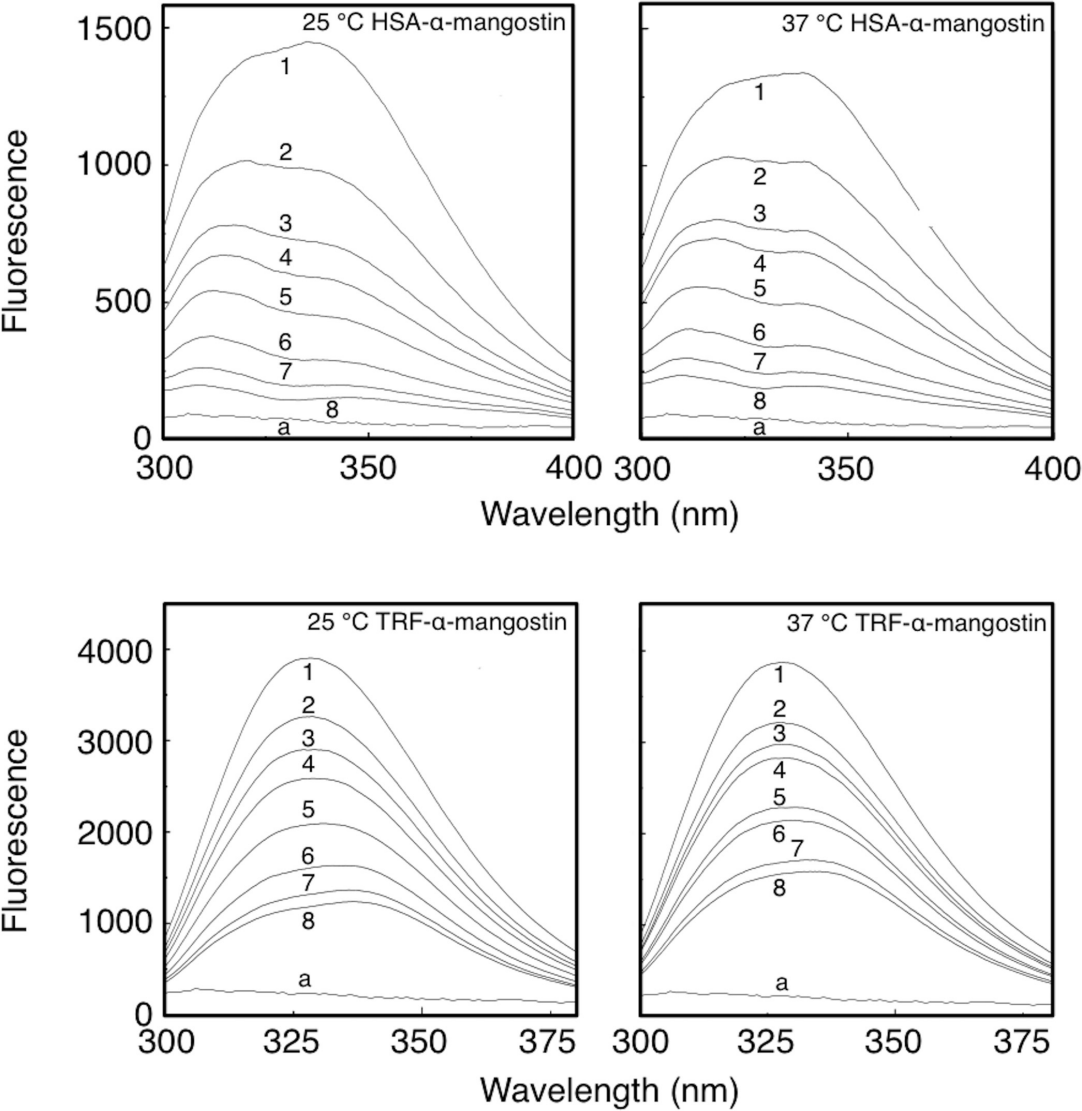
Fluorescence quenching spectra of α-mangostin-HSA and α-mangostin-TRF systems at 25°C and 37°C. *C*_protein_ = 10 μM; *C*_α-mangostin_ 1 to 8 = 0, 4, 8, 10, 16, 24, 32, 40 μM. Spectrum a: 40 μM α-mangostin with no protein added.

Fig 3 is showing the fluorescence quenching spectra of the α-mangostin-protein complex at different concentrations of α-mangostin at 25°C or 37°C at a fixed concentration of HSA or TRF (10 μM).

It can be seen from Fig 3 that the fluorescence intensity of HSA or TRF decreased with increasing concentration of α-mangostin. A blue- and red-shift in the maximum emission wavelength of fluorescence was observed for HSA-α-mangostin and TRF-α-mangostin, respectively, as summarized in Table 3. In Fig 3, the fluorescence spectrum of α-mangostin (curve a) is showing that α-mangostin exhibits no fluorescence, Consequently, addition of α-mangostin will not produce any fluorescence that interfere with HSA or TRF fluorescence, which indicated that an interaction between α-mangostin and HSA or TRF took place, which changed the microenvironment of the proteins. Combined with the results given in Table 3, we may conclude that α-mangostin increased the hydrophobic microenvironment of HSA and decreased the hydrophobic microenvironment of TRF. Because the two proteins have different structure, the binding mechanism of α-mangostin and protein and the degree of change of the microenvironment of the proteins were different.

**Table 3:** The displacement of greatest emission peak of α-mangostin-HSA/TRF system.

System	shifts of maximum emission wavelength	
	298 K	310 K
HSA-α-mangostin	blue shift 27 nm	blue shift 29 nm
TRF-α-mangostin	red shift 9 nm	red shift 6 nm

It can be seen from Fig 3 that at an excitation wavelength of 282 nm, the maximum emission wavelength is 335 nm and 328 nm for HSA and TRF, respectively, and from the ultraviolet absorption spectra of α-mangostin, it can be concluded that α-mangostin is absorbing at the excitation wavelength of 282 nm and at the emission wavelength of different proteins between 328 and 335 nm. Therefore, the filter effect should be considered in this study. The formula (2) was used to correct the filter effect.

$$F_{corr}=F_{obs}x10^{A\left(\lambda_{exc}\right)/2}x10^{A\left(\lambda_{em}\right)/2}$$ (2)

*F*_corr_ is the value of fluorescence after correction, *F*_obs_ is the measured value of fluorescence, *A*(λ_exc_) is the absorption value at the excitation wavelength, *A*(λ_em_) is the absorption value at the emission wavelength. All the values of fluorescence intensity were corrected in this study.

Mechanisms of fluorescence quenching are usually classified into dynamic quenching and static quenching. The dynamic fluorescence quenching conforms to the Stern-Volmer equation. In order to determine the mechanism of fluorescence quenching between α-mangostin and serum protein, the system mentioned above could be analyzed by the Stern-Volmer equation:

$$F_{0}/F=1+K_{q}\tau_{0}\left[D\right]=1+K_{SV}\left[D\right]$$ (3)

*F*_0_ and *F* are fluorescence intensities of the biomacromolecule in the absence and presence of added quencher, respectively; [*D*] is the concentration of the quencher; *K*_q_ is the rate constant of dimolecular quenching; *τ*_0_ is the average lifetime of the biomacromolecule without quencher, mol/L; *K*_sv_ is the quenching constant of Stern-Volmer, L/mol (*K*_q_ = *K*_SV_/*τ*_0_).

**Fig 4:**
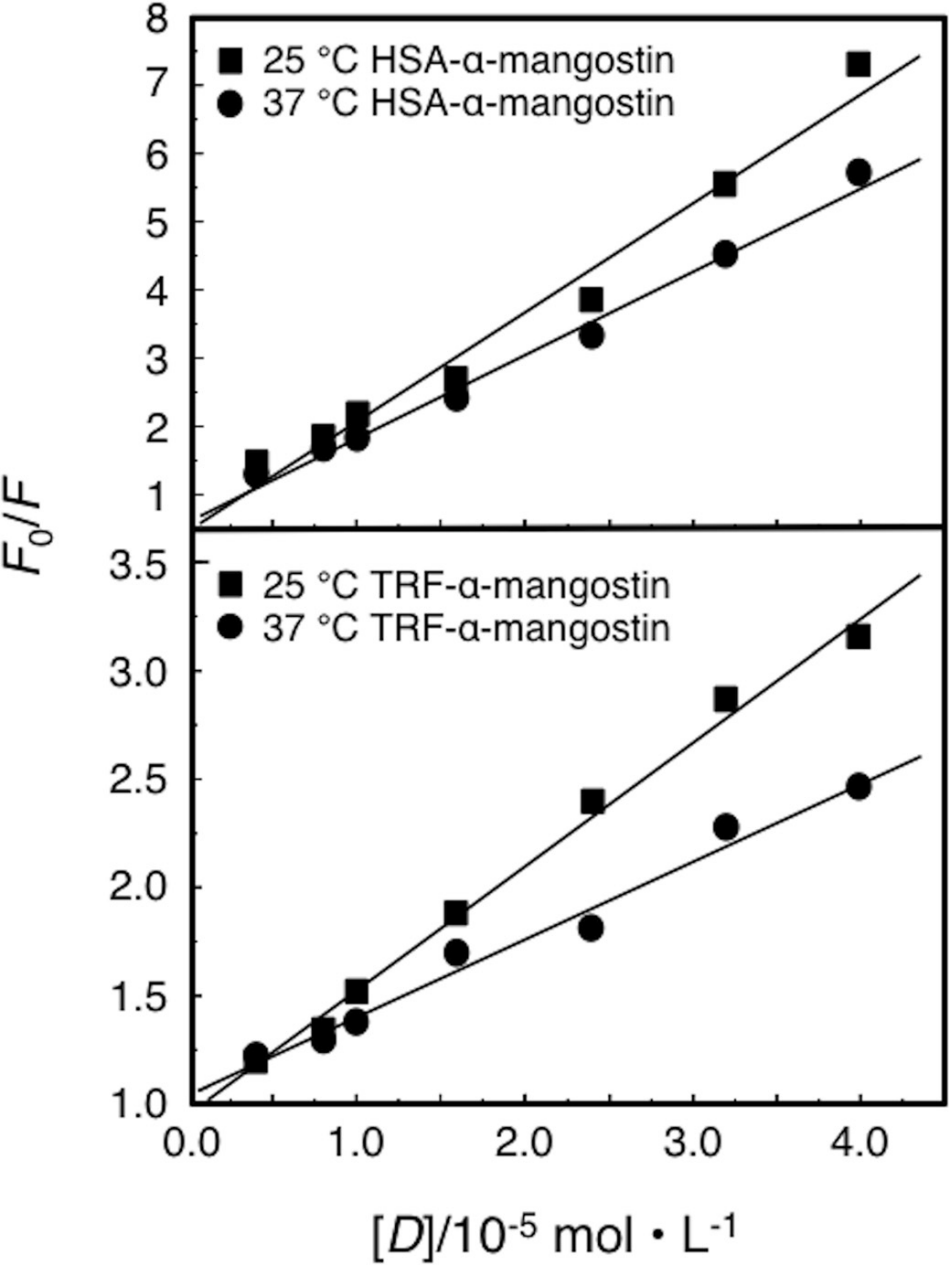
Stern-Volmer plot for the fluorescence quenching of HSA and TRF by α-mangostin.

Stern-Volmer plots of the fluorescence quenching between HSA or TRF and α-mangostin are shown in Fig 4 and the calculated quenching constants are given in Table 4.

**Table 4:** The quenching constants that between α-mangostin and HSA/TRF.

Protein	298 K		310 K	
	*K*_SV_ (L/mol)	*K*_*q*_ (L/mol)	*K*_SV_ (L/mol)	*K*_*q*_ (L/mol)
HSA	1.5962×10^5^	1.5962×10^13^	1.2165×10^5^	1.2165×10^13^
TRF	0.5724×10^5^	0.5724×10^13^	0.3589×10^5^	0.3589×10^13^

By the variable temperature experimental method, we could directly determine the fluorescence quenching mechanism. If the temperature effect is negative, the quenching is static quenching; if the temperature effect is positive, the quenching is dynamic [26–29]. It was found that the value of *K*_q_ dropped significantly with increasing temperature (Table 4), which proved that the quenching between HSA/TRF and α-mangostin is static. Another method to show static quenching is to compare the rate constant of dimolecular quenching with the maximum scatter collision quenching constant. The measured *K*_*q*_ is three orders of magnitude higher than the maximum scatter collision quenching constant (2.0×10^10^ L·mol^-1^s^-1^), indicating a static quenching mode for α-mangostin upon the binding of HSA and TRF.

### The binding constant and binding sites between α-mangostin and HSA and TRF

The binding constant (*K*) between biomacromolecule and quenching molecules may be calculated by Eq (4) [30,31]

$$\mathrm{lg}\left[\left(F_{0}-F\right)/F\right]=\mathrm{lg}K+n\mathrm{lg}\left[D_{t}\right]$$ (4)

**Fig 5:**
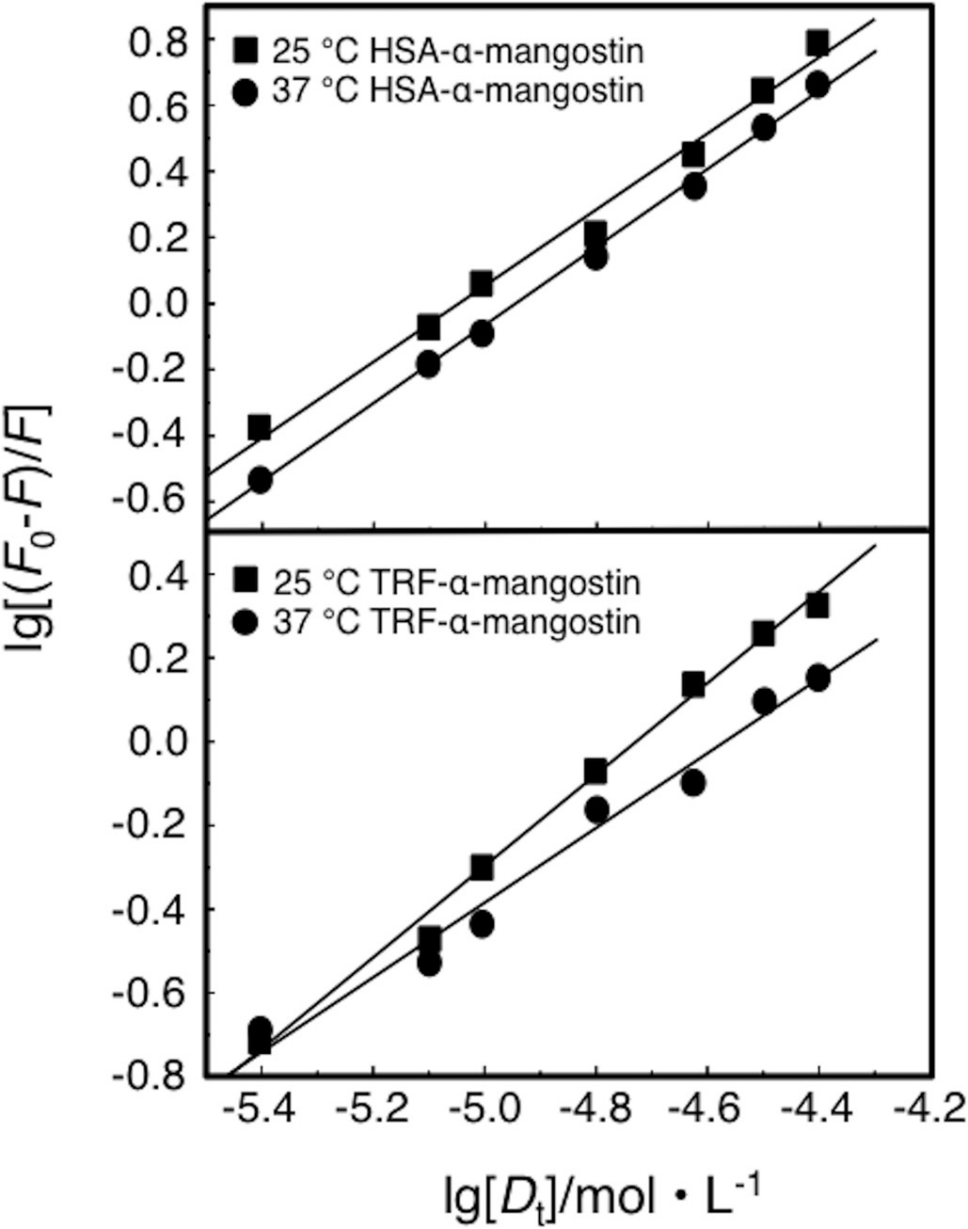
Plot of lg[(*F*_0_-*F*)/*F*] and lg[*D*_*t*_] of α-mangostin-HSA and α-mangostin-TRF.

*F*_0_ and *F* are fluorescence intensities of biomacromolecule in the absence and presence of the quencher, respectively; [*D*_*t*_] is the concentration of the quencher; *K* is the binding constant, L/mol; *n* is the number of binding sites. Combining the experimental data with Eq (4) by drawing double logarithmic plots of lg[*D*_t_] and lg[(*F*_0_-*F*) /*F*] for each binding reaction system, resulted in plots as shown in Fig 5. The calculated results including the binding constants between α-mangostin and HSA/TRF are summarized in Table 5.

**Table 5:** The binding constant of α-mangostin binding with HSA/TRF.

Protein	T/K	fitted equation	*SD*	*N*	R (L·mol^-1^·s^-1^)	*K* (L·mol^-1^)	*N*
HSA	298	*y* = 5.8118+1.1517*x*	0.0453	7	0.9950	6.4832×10^5^	1.15
	310	*y* = 5.8955+1.1934*x*	0.0215	7	0.9989	7.8619×10^5^	1.19
TRF	298	*y* = 5.1659+1.0929*x*	0.0342	7	0.9968	1.4652×10^5^	1.09
	310	*y* = 4.0638+0.8897*x*	0.0510	7	0.9895	1.1582×10^5^	0.89

Table 5 shows; (1) The molecule binding constants of drug and serum protein are large, and there is around one binding site, which indicated that α-mangostin and HSA/TRF formed relatively stable complexes, which confirmed that the fluorescence quenching mechanism is of the static type; (2) The structural differences of the two protein molecules studied are reflected in the binding constant, the complex formation of α-mangostin and HSA is relatively strong; (3) The temperature has an influence on the binding constant and binding sites of drug-protein system. With increasing temperature, the binding constant and number of binding sites of the α-mangostin/HSA system increased while the binding constant and number of binding sites of the α-mangostin/TRF system decreased.

### Determine the parameter of energy transfer between α-mangostin and HSA/TRF

The intermolecular energy transfer efficiency of drug-protein interactions and distance betwen fluorescence emission residues in drug-protein molecules may be calculated according to Fōrster energy transfer theory [32,33]. The energy transfer efficiency *E* is dependent on the binding distance r and the critical energy transfer distance R_0_ as shown in Eq (5):

$$E=R_{0}^{6}/\left(R_{0}^{6}+r^{6}\right)$$ (5)

Where *E* is the transfer efficiency, and *R*_0_ is the critical energy transfer distance when *E* = 50%.

$$R_{0}^{6}=8.8x10^{-25}K^{2}N^{-4}\Phi J$$ (6)

Where *K*^2^ is the dipole spatial orientation factor (*K*^2^ = 2/3); *N* is the refractive index of the medium (*N* = 1.336); *Φ* is the light quantum efficiency (*Φ* = 0.13) of the donor (protein) [33,34] and *J* is the overlap integral between fluorescence emission spectrum of the donor and absorption spectrum of the acceptor (drugs), cm^3^∙L/mol. *J* can be described as:

$$J=\int_{0}^{\infty }F\left(\lambda \right)\varepsilon \left(\lambda \right)\lambda^{4}d\lambda /\int_{0}^{\infty }F\left(\lambda \right)d\lambda$$ (7)

*F*(λ) is the fluorescence intensity of the fluorescence donor at the wavelength λ and *ε*(λ) is the molar absorption coefficient of the acceptor at the wavelength λ, L/(mol·cm).

The energy transfer efficiency *E* can be calculated by Eq (8):

$$E=1-F/F_{0}$$ (8)

**Fig 6:**
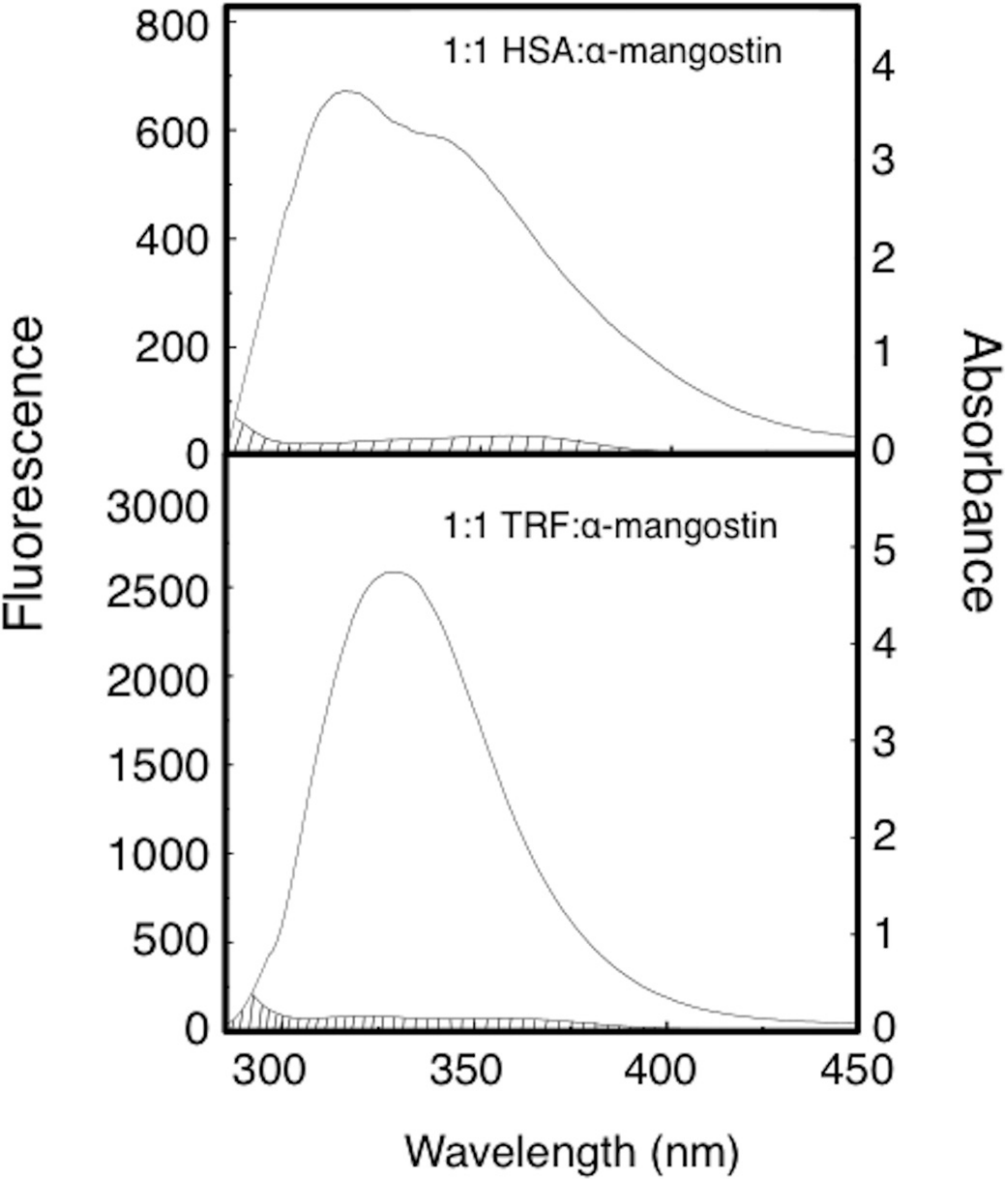
The fluorescence spectrum and absorption spectrum overlap charts of the binding reaction between α-mangostin and HSA or TRF.

According to the experimental results, the fluorescence overlap integral *J* of the fluorescence emission spectrum of HSA/TRF and the absorption spectrum of α-mangostin-HSA/TRF may be calculated by Eq (7). The critical energy transfer distance *R*_0_ may be calculated by substitution of *J* into Eq (6). The energy transfer efficiency *E* of every system may be calculated by Eq (8), and the binding distance *r* of every system may be calculated by introducing *E* into Eq (5). The fluorescence spectra and absorption spectra overlap charts of the binding reaction between α-mangostin and HSA/TRF are shown in Fig 6 and the calculated corresponding energy transfer parameters are shown in Table 6.

**Table 6:** The energy transfer parameters between α-mangostin and HSA and TRF.

Protein	*J* (cm^3^·dm^3^·mol^-1^)	*R*_*0*_ *(nm)*	*E*	*R (nm)*
HSA	2.07×10^−14^	2.78	0.54	2.71
TRF	1.77×10^−14^	2.71	0.34	3.03

From Table 6 we can conclude that the binding distance between α-mangostin and HSA or TRF is less than 7 *nm*, indicating that non-radiative energy transfer occurred between the drug and HSA or TRF.

The following conclusions may be drawn by an analysis: (1) The differences in molecular structure between HSA and TRF led to a higher energy transfer efficiency (*E*) in the HSA-α-mangostin system than in the TRF-α-mangostin system. The binding distance (*r*) is smaller in the HSA-α-mangostin system than in the TRF-α-mangostin system, which indicates that the HSA-α-mangostin system is more stable. These results are in accordance to the results of the fluorescence quenching experiment described above. (2) The transfer efficiency (*E*) of the HAS-α-mangostin and TRF-α-mangostin systems was high and the binding distance of both systems was about 3 nm.

### The synchronous fluorescence spectra of α-mangostin and HSA/TRF

**Fig 7:**
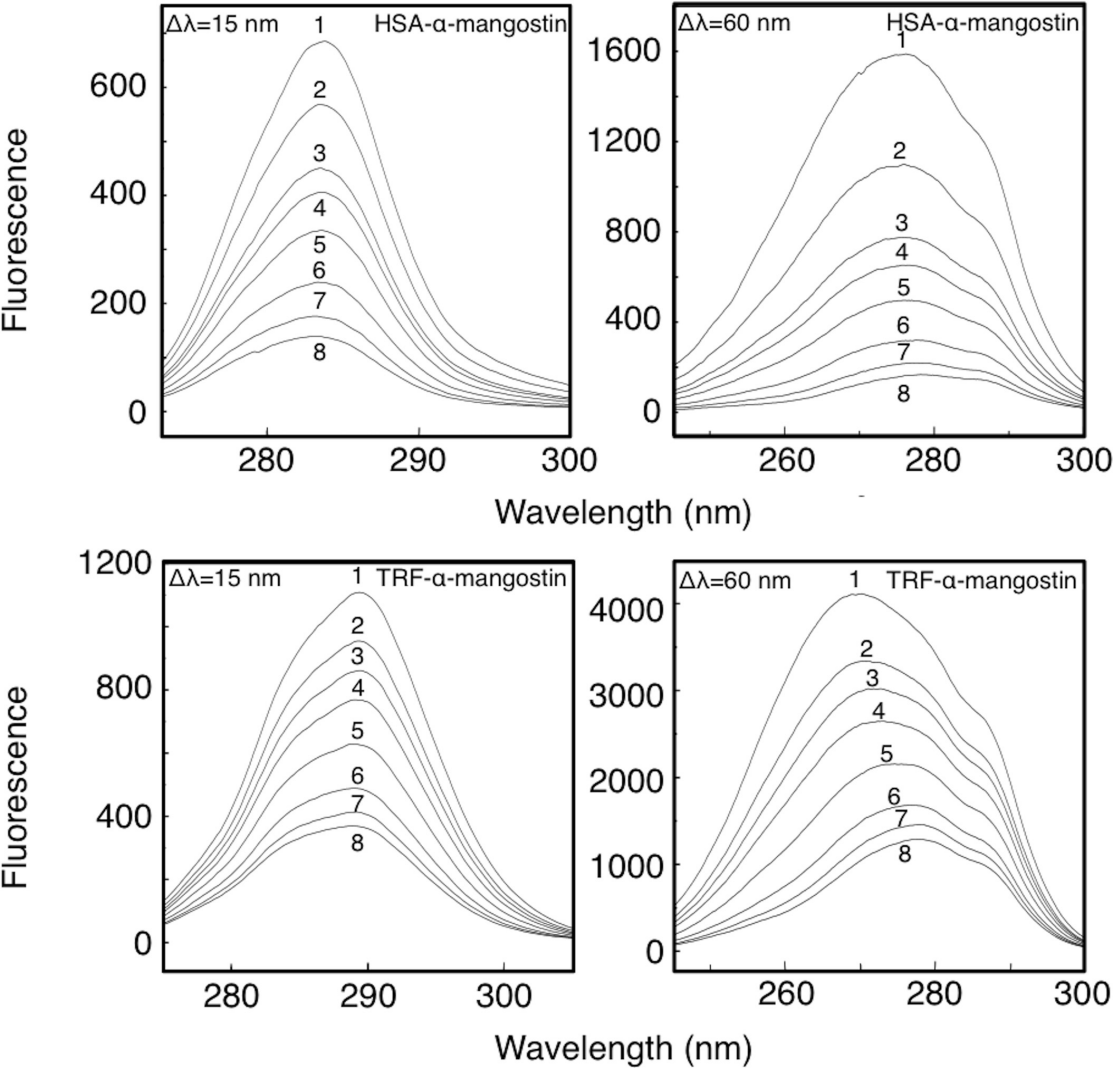
Effect of α-mangostin on synchronous fluorescence spectra of HSA or TRF. *C*_protein_ = 10 μM; *C*_α-mangostin_ 1 to 8 = 0, 4, 8, 10, 16, 24, 32, 40 μM.

Synchronous fluorescence spectra can accurately distinguish the system in which excitation and emission spectra are overlapping and give information on the influence of drug molecules on protein conformation. In synchronous fluorescence spectra, a Δλ = 15 nm shows the presence of tyrosine residues and a Δλ = 60 nm shown the presence of tryptophan residues. The displacement of maximum fluorescence emission peak of amino acid residues reflects the change of the polarity in the microenvironment. The synchronous fluorescence spectra of α-mangostin and HSA or TRF are shown in Fig 7 and the displacements of maximum fluorescence emission peak of α-mangostin-HSA or TRF systems are shown in Table 7.

**Table 7:** The displacements of maximum fluorescence emission peak of HSA-α-mangostin and TRF-α-mangostin.

System	The displacement of greatest emission peak	
	Δλ = 15 *nm*	Δλ = 60 *nm*
HSA-α-mangostin	blue shift 0.4 nm	red shift 2 nm
TRF-α-mangostin	blue shift 0.6 nm	red shift 8 nm

The results obtained (Fig 7 and Table 7) were: (1) when the concentration of serum protein was fixed, characteristic fluorescence spectrum peaks of tyrosine or tryptophan residues were quenched with increasing concentrations of α-mangostin, and the drop of fluorescence intensity of tryptophan residues was much greater than that of tyrosine residues, suggesting that drugs and tryptophan residues in protein were more likely to interact. (2) With increasing concentration of α-mangostin, the characteristic fluorescence spectrum peak of tyrosine residues of the serum proteins had a weak blue shift, while the characteristic fluorescence spectrum peak of tryptophan residues of the serum proteins exhibited a significant red shift, indicating that after binding of α-mangostin to the serum proteins, the protein conformation was changed; the environment polarity of tyrosine residues was slightly decreased and its hydrophobicity slightly increased. But the environment polarity of tryptophan residues increased significantly while the hydrophobicity was significantly reduced. The influence of α-mangostin on the microenvironment of tryptophan residues of the serum proteins was more obvious. (3) As the molecular structural differences between HSA and TRF, the displacement extent of the maximum emission wavelength of synchronous fluorescence spectra of TRF caused by α-mangostin was greater than that of HSA at Δλ = 15 nm and Δλ = 60 nm. It indicated that the degree of change of the hydrophobic microenvironment of tyrosine and tryptophan residues in TRF molecule caused by α-mangostin was greater than that observed for HSA.

### The effect on microenvironment of serum protein tryptophan caused by α-mangostin

Analysis of the results of the synchronous fluorescence spectra above suggests that the possibility of α-mangostin interacting with tryptophan residues in serum proteins is high and that α-mangostin may have a great influence on the micro zone structure of tryptophan residues. The polarized fluorescence spectrum can reflect this effect. Polarized fluorescence spectrum was measured by the degree of polarization *P* and anisotropy *r*, [35–38] *P* may be calculated by the formula:

$$P=\left(I_{VV}-GI_{VH}\right)/\left(I_{VV}+GI_{VH}\right)$$ (9)

Where *G* is the correction factor (*G* = *I*_*HV*_/*I*_*HH*_), *I*_VH_ and *I*_VV_ are the vertically polarized emission intensity and horizontally polarized emission intensity, respectively, under the polarized light of the vertical excitation. *I*_HV_ and *I*_HH_ are the vertically polarized emission intensity and horizontally polarized emission intensity, respectively, under the polarized light of the horizontal excitation.

Anisotropy *r* may be calculated by the following formula:

$$r=\left(I_{VV}-I_{VH}xG\right)/\left(I_{VV}+2I_{VH}xG\right)$$ (10)

The fluorescence polarization parameters of α-mangostin-HSA and TRF systems are summarized in Table 8.

**Table 8:** The fluorescence polarization parameters of α-mangostin-HSA/TRF systems.

System	*I*_VV_	*I*_VH_	*I*_HV_	*I*_HH_	*G*	*P*	*r*
HSA	95.9800	71.8900	57.5400	55.9100	1.0292	0.1294	0.0902
HSA-α-mangostin	58.8800	38.8100	31.1600	30.0900	1.0356	0.1887	0.1342
TRF	232.1000	174.7000	139.3000	132.4000	1.0521	0.1161	0.0805
TRF-α-mangostin	154.3000	116.8000	93.1900	89.0300	1.0467	0.1159	0.0803

The degree of polarization of phosphor was connected with rotational speed of the fluorescent molecule; the faster the rotational speed, the smaller the fluorescence polarization; the slower the rotational speed, the greater the fluorescence polarization [39]. From Table 8, the following results can be obtained: (1) Fast rotational speed of the serum protein in buffer solution led to a small degree of polarization *P* and a small anisotropy *r*. Due to the differences of the molecular structure of the two proteins, the rotational speed of TRF in buffer solution was faster than that of HSA, which was reflected in a lesser degree of polarization *P* and anisotropy *r* of TRF compared to that of HSA. (2) After adding the drug, α-mangostin combined with the serum proteins. If the bonding is tight, the fluorescence molecules will have long relaxation time and the degree of fluorescence polarization *P* will be high; conversely, if the bonding is weak, the fluorescence molecules will have short relaxation time and the degree of fluorescence polarization *P* will be low. After complex formation with the drug, the degree of polarization *P* and anisotropy *r* of TRF became lower, indicating that the complex generated from the binding of α-mangostin to TRF had short relaxation time and the bonding was weak. While after complex formation with the drug, the degree of polarization *P* and anisotropy *r* of HSA became higher, indicating that the complex generated from the binding of α-mangostin to HSA exhibited a long relaxation time and the bonding was tight. Generally speaking, as for the α-mangostin-HSA or -TRF system, the fluorescence polarization experiment reconfirmed that α-mangostin and HSA or TRF could interact with each other, which affected the conformation of the protein. The complex between α-mangostin and HSA was stronger than that of TRF and it was consistent with the results obtained from the fluorescence spectra above.

### Determine the interaction between α-mangostin and HSA or TRF

The main interaction between small drug molecules and proteins are hydrophobic interactions, hydrogen bonds and van der Waals forces, electrostatic forces, etc [40]. The interaction may be different between different types of small drug molecules and protein macromolecules with different structures [41] summerized the law of thermodynamics, which may indicate the type of interaction between small drug molecules and biological macromolecules based on extensive experimental results. The following thermodynamic parameters can roughly determine the type of interaction of α-mangostin with HSA or TRF. The binding reaction between drug and protein can occur spontaneously when Δ*G* < 0; hydrophobic interaction exists in the α-mangostin-HSA/TRF system when Δ*H* > 0 and Δ*S* > 0; hydrogen bonds and van der Waals forces exist in the α-mangostin-HSA/TRF system when Δ*H* < 0 and Δ*S* < 0; mainly electrostatic attraction exists in the α-mangostin-HSA/TRF when Δ*H* < 0 and Δ*S* > 0 [40].

According to Eqs (11), (12), (13) and the binding constant *K* that has been calculated, we could calculate the thermodynamic parameters of the binding reaction between α-mangostin and serum proteins and the calculated thermodynamic parameters are listed in Table 9.

ΔG=ΔH−TxΔS (11)

ΔG=−RTlnK (12)

$$\ln\left(K_{2}/K_{1}\right)=\left(\Delta H/R\right)x\left(1/T_{1}-1/T_{2}\right)$$ (13)

Table 9 shows that the determined Δ*G* of the two systems is negative, indicating a spontaneous binding process between α-mangostin and HSA/TRF. HSA-α-mangostin has a smaller Δ*G*, indicating that HSA binds stronger than TRF to α-mangostin. The TRF-α-mangostin complex exhibits negative Δ*H*- (-162 kJ/mol) and Δ*S*-values (-442 kJ/mol), suggesting that van der Waals forces and hydrogen bonding play major roles in this binding [41,42]. However, the HSA-α-mangostin complex exhibits positive Δ*H-* (12.3 kJ/mol) and Δ*S*-values (153 kJ/mol), indicating the dominant role of hydrophobic forces during this binding process. The observed distinct different binding of α-mangostin is mainly due to structural differences of HSA and TRF.

**Table 9:** The thermodynamic parameters of α-mangostin binding with HSA/TRF.

System	298K			310K		
	Δ*H* (*k*J/mol)	Δ*G* (*k*J/mol)	Δ*S* (J/mol·K)	Δ*H* (*k*J/mol)	Δ*G* (*k*J/mol)	Δ*S* (J/mol·K)
HSA- α-mangostin	12.3	-33.2	153	12.3	-35.0	153
TRF- α-mangostin	-162	-29.4	-446	-162	-24.2	-446

### Study of molecular modeling

**Fig 8:**
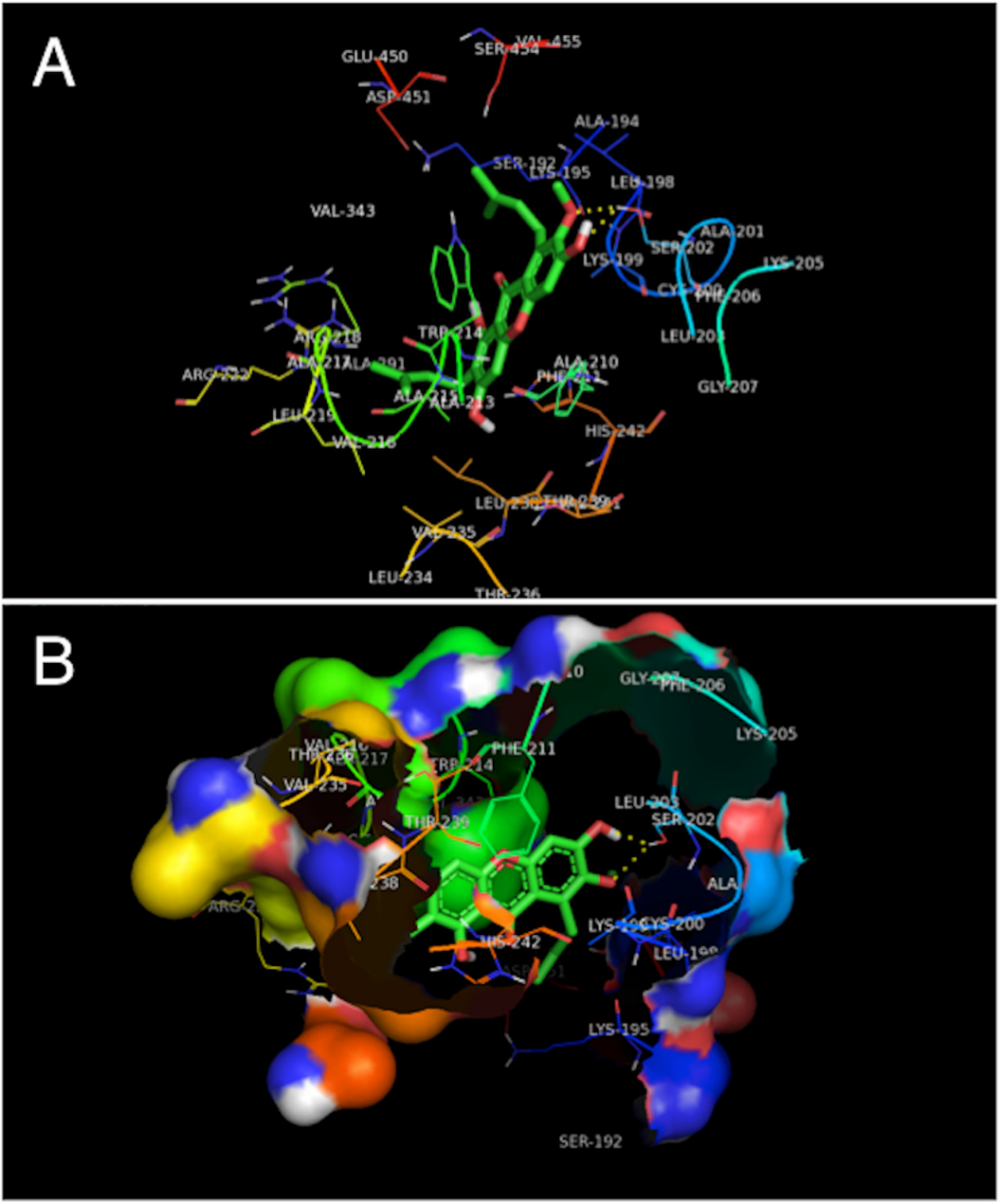
**Interaction mode (A) and hydrophobic surface map (B) between α-mangostin and HSA.** Only residues within 10.0 Å of the ligand are displayed. The residues of HSA are represented using line model, and the ligand structure are represented using stick model. The hydrogen bonds between the ligand and the protein are represented by yellow dashed lines.

**Fig 9:**
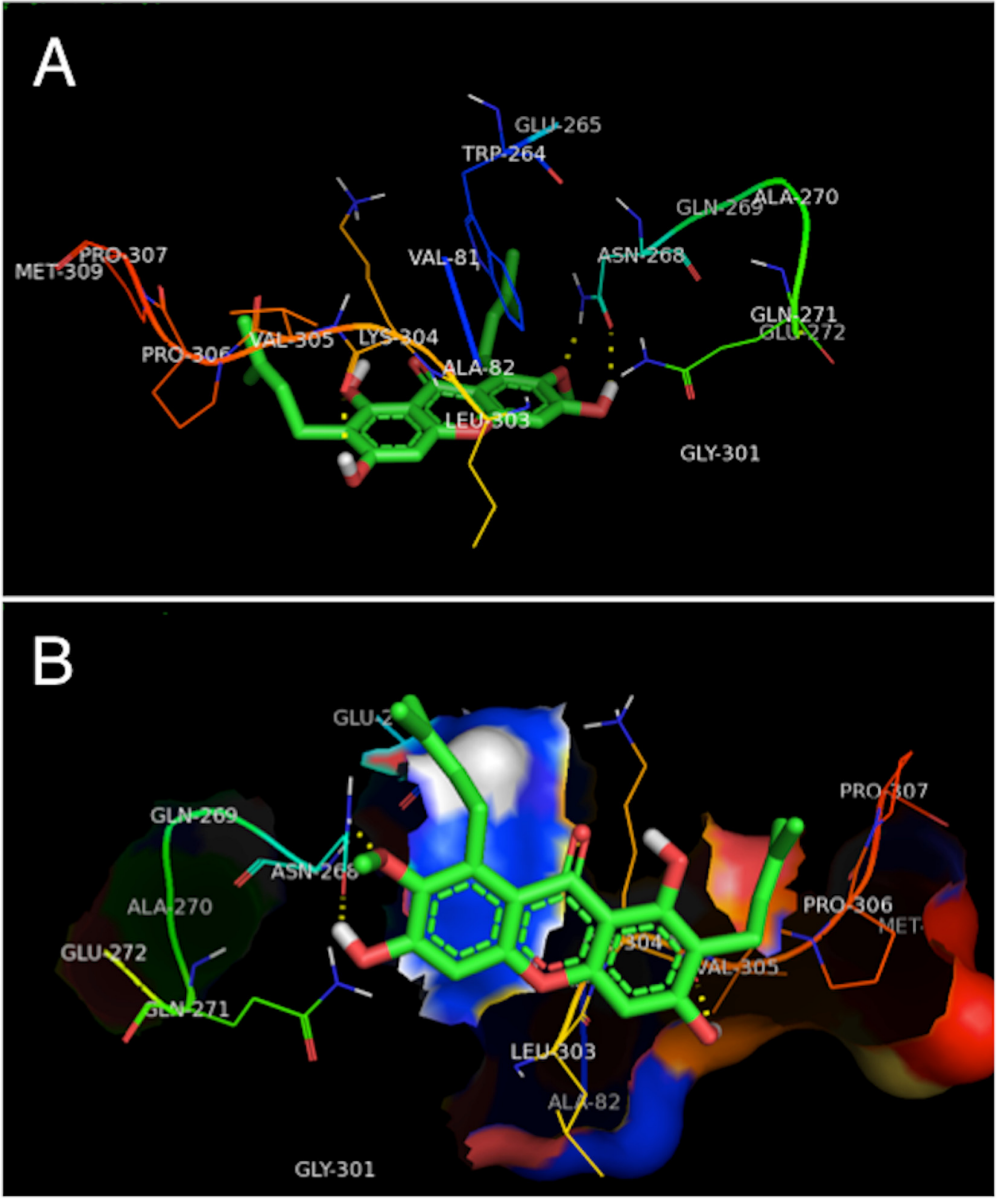
**Interaction mode (A) and hydrophobic surface map (B) between α-mangostin and TRF.** Only residues within 10.0 Å of the ligand are displayed. The residues of TRF are represented using line model, and the ligand structure are represented using stick model. The hydrogen bonds between the ligand and the protein are represented by yellow dashed lines.

The molecular docking method offers a web-based, easy to use interface that handles all aspects of molecular docking from ligand and protein set-up. Molecular docking integrates a number of computational chemistry software specifically aimed at correctly calculate parameters needed at different steps of the docking procedure, *i*.*e*. accurate ligand geometry optimization, energy minimization, charge calculation, docking calculation and protein-ligand complex representation. Thus, the use of molecular docking allows the user to carry out highly efficient and robust docking calculations by integrating a number of popular software used in *in silico* chemistry into one comprehensive web service. Molecular docking is established based on experimental methods, and the molecular docking method is only one way. The results of molecular docking are verified by experiment [43]. In this study, experiments and docking studies have been carried out, and the molecular docking results indicate a high incorporation of the spectrum experiments. Although molecular docking has shortcomings, it has been used widely [43–46] and molecular docking can be used as an auxiliary method. The interaction and binding site between drugs and protein may be specifically shown by computer molecular simulation [43]. The model of binding interaction between α-mangostin and HSA/TRF was built by molecular docking. The structure of HSA was obtained from PDB crystal database, numbering lh9z, the only tryptophan residue in HSA molecule is Trp214 (located in domain II). Considering the characteristics of molecular structure of α-mangostin, in the process of molecular docking, the warfarin binding pocket was considered as the active site. The crystal structure of TRF was also obtained from PDB crystal database, numbering 1D4N. The optimal combination model of α-mangostin binding with HSA/TRF was obtained in accordance with the best energy principle. The molecular docking of α-mangostin to HSA is complicated by the fact that this protein carries to drug binding sites, Sudlow's site I and site II. Therefore, the established molecular docking system of the protein active site and α-mangostin was verified by re-docking of the ligand. The potential energy function was used as the evaluation function to find the best combination of ligand and receptor. The evaluation function determined that the Sudlow 's site II is the active site between α-mangostin with HSA. The fluorescence quenching experiment showed that the ligand directly affects the tryptophan residues, so α-mangostin was automatically docked into the binding cavity of TRF carrying the tryptophan residue. Using the evaluation function it was possible to establish the best active site. The results of molecular simulation systems are shown in Figs 8 and 9.

Fig 8 shows α-mangostin is located in the active pocket of HSA and the whole molecule is surrounded, free energy of binding *E* = -8.71 *k*cal/mol. The distance between α-mangostin and HSA tryptophan residues (Trp214) and phenylalanine residues (Phe206, Phe211) is small, which provides convincing evidence that α-mangostin has fluorescence quenching effect on the HSA molecule. In the process of α-mangostin molecule binding to HSA, two hydrogen bonds involving Ser202 with 7-OCH_3_ of α-mangostin and Ser202 with 6-OH of α-mangostin are formed. In addition, non-covalently bound α-mangostin is at the active site of HSA and a hydrophobic area around its binding pockets is mainly made up of Trp214, Phe206, Phe211, Ala194, Ala210, Ala213, Ala215, Ala217, Ala291, Leu198, Leu203, Leu219, Leu234, Leu238, Val216, Val235, Val241, Val343, and Val455. This indicates that the hydrophobic interaction at the binding site between α-mangostin and HSA is the main driving force of the binding. From the results of molecular simulation, we may conclude that the main interaction between α-mangostin and HSA is hydrophobic interaction but also that hydrogen bonds exist, which is consistent with the results of the thermodynamic experiment.

Fig 9 shows α-mangostin is located in the active pocket of TRF and the whole molecule is surrounded, free energy of binding *E* = -6.94 *k*cal/mol. The distance between α-mangostin and TRF tryptophan residues (Trp264) is small, which provides convincing evidence that α-mangostin has fluorescence quenching effect on the TRF molecule. In the process of α-mangostin molecule binding to TRF, three hydrogen bonds involving Lys304 with 3-OH of α-mangostin, Asn268 with 6-OH of α-mangostin and Asn268 with 7-OCH_3_ of α-mangostin are formed. In addition, non-covalently bound α-mangostin is at the active site of TRF and a hydrophobic area around its binding pocket is mainly made up of Trp264, Ala 82, Ala 270, Leu303, Val81, Val305, Pro306, Pro307, and Met309. This indicates that hydrophobic interactions at the binding site exist between α-mangostin and TRF. From the results of molecular simulation, we may conclude that the main interactions between α-mangostin and TRF are hydrogen bonds but also that hydrophobic interaction exists, which is consistent with the results of the thermodynamic experiment.

Comparing Figs 8 and 9, it is evident that the degree of integration of α-mangostin in the active site of HSA is significantly greater than that of α-mangostin in TRF, due to differences in protein structure. Furthermore, the binding mechanism of α-mangostin-HSA is different from the one of α-mangostin-TRF, which is consistent with the results of spectral experiment above.

## Conclusions

In this paper, α-mangostin of relatively high purity was extracted successfully using macroporous resin. The extraction process engineering is simple, inexpensive with high yields. The reaction mechanism between α-mangostin and HSA/TRF has been studied by fluorescence and UV spectroscopy, as well as by computer molecular simulation. We calculated binding, energy transfer efficiency and thermodynamic parameters and results of molecular docking of reaction systems. The experimental results show that the fluorescence quenching mechanism between α-mangostin and HSA/TRF were both static quenching and the binding distances between α-mangostin and HSA/TRF were both less than 7 *nm*, indicating that an energy transfer exists between the α-mangostin and HSA/TRF. After comprehensively comparing the K_a_-value with the K_sv_-value, we found that the free binding energy during docking is consistent with the stability of the α-mangostin-HSA complex as determined by spectroscopy. The *K*_sv_-value reflects the strength of drug interaction with the proteins, while the spectroscopically determined *K*_a_-value indicates the binding strength between α-mangostin and HSA/TRF. The determined K_sv_-value is consistent with the *K*_a_-value obtained. The *K*_a_-value is closely related to the free binding energy during docking; the greater the *K*_a_ value, the smaller the free binding energy, which indicates increased stability of the α-mangostin-HSA/TRF complex. According to current literature, drug binding alters protein conformation. In general, there are three commonly used methods to investigate this, *i*.*e*. the detection by the CD spectrum, the attenuated total reflection infrared spectrum (ATR-IR), and the fluorescence spectrum [44–46]. In this work, fluorescence spectrum experiments, synchronous fluorescence experiments and fluorescence polarization experiments were used to investigate protein conformation. We can conclude that the drug molecule changes the protein conformation and the hydrophobicity of the tryptophan microenvironment. The differences of protein structure caused differences in the degree of change and we found that the bonding strength of the α-mangostin-HSA system was greater than that of the α-mangostin-TRF system.

Combining thermodynamic experiments with the molecular modeling studies, we obtained binding domains, binding sites of non-covalent interaction, amino acid residues, mode of interaction information and intuitive combination reaction models of α-mangostin with serum protein. Relevant results can be the theoretical reference to study pharmacological mechanism of α-mangostin.

## Experimental Section

### Materials and instruments

Mangosteen fruits were collected at the Tianmushan nature reserve, Lin’an, Zhejiang province, China. Tianmushan nature reserve is a national nature reserve of China where the plants are state-owned. Zhejiang Agricultural & Forestry University is a state-owned public university of the Zhejiang province, China, and Zhejiang Agricultural & Forestry University has built practice teaching facilities in the Tianmushan nature reserve. The natural resources of the teaching practice base can be used as experimental materials, and mangosteen fruits are available in the reserve. Mangosteen is not an endangered or protected species, so it can be used. The picking of mangosteen fruits in this reserve was approved by the academic committee of Zhejiang Agricultural & Forestry University and our mangosteen experiment conformed with the wild plants protection regulation of the People's Republic of China; α-Mangostin standard (≥98%) was supplied by Shanghai Chunyou Biological Technology Co., Ltd., China; Isopropyl alcohol, methanol and ethanol were all HPLC grade and obtained from Shanghai Xingke High Purity Solvent Co., Ltd., China; Deuterated chloroform (≥99.96%) was from Aladdin reagent, Shanghai Co., Ltd., China and HPD-400 macroporous resin from Zhengzhou Qinshi Technology Co., Ltd., China; Human serum albumin (HSA, ≥98%), human transferrin (TRF, ≥98%), tris(hydroxmethyl) aminomethane (Tris, GR) were all purchased from Shanghai Huamei Biological Engineering Company, China; Hydrochloric acid and sodium chloride were analytical reagent from J&K Scientific Ltd., Shanghai, China. Deionized water with sub-boiling distillation was used in all experiments. The following solutions were prepared: 0.1 M Tris-HCl buffer, pH = 7.3, containing 0.1 M NaCl to maintain ionic strength; HSA, TRF and mixed protein solution (with different volume ratio of HSA and TRF) with the concentration of 10 μM using the buffer solution; 1.0 mM α-mangostin stock solution.

The following equipments were used: GC/MS-QP2010SE gas chromatograph-mass spectrometer (GC-MS) (Shimadzu, Japan); IR Prestige-21 Fourier transform infrared spectrometer (IR) (Shimadzu, Japan); FW-4 tablet press (Tianjin Optical Instrument Factory); AVANCE II /400MHz nuclear magnetic resonance (NMR) (Bruker, Switzerland); F-7000 fluorophotometer (Hitachi Company, Japan); UV-2550 ultraviolet-visible spectrophotometer (UV-VIS) (Shimadzu, Japan); Agilent 1100 high performance liquid chromatograph (HPLC, Agilent company, America); ZD-2 precision acidity meter (Shanghai Leici Instrument Factory).

### Experimental methods. The isolation, separation and structural identification of α-mangostin

The HPD-400 macroporous resin was soaked in 95% ethanol for several hours. Then it was rinsed with a large volume of distilled water. Using the wet method, the cleaned resin was packed into the chromatography column with stirring. After the resin had sedimented, the water was drained off and subsequently the resin was washed with 95% ethanol at the rate of 3 column volumes per hour (V/h) until the effluent had no white turbidity when distilled water was added. Subsequently, the resin was washed with distilled water at the same flow rate.

HPLC was carried out using an Agilent 1100 Eclipse XDB—C_18_ chromatographic column (250 mm× 4.6 mm, 5 μm, Agilent Company, America) under the following conditions: column temperature: 30°C; mobile phases: methanol (A) and water (B); flow rate: 0.5 mL/min; detection wavelength: 317 nm; sample size: 5 μL; elution conditions: 0–30 min used A of 88%, 30–60 min used A from 88% to 70%, 60–120 min used A from 70% to 80%.

Dry native mangosteen pericarps (100 g) were crushed with a shredder. To extract α-mangostin, the pericarp preparation obtained was extracted at 40°C three times with 70% isopropyl alcohol (800 mL, 600 mL and 400 mL, respectively). The combined extract was reduced to 305 g by evaporating isopropyl alcohol. Purified water (2000 ml) was used to disperse the extract. The dispersed extract was passed over the preprocessed HPD-400 macroporous resin. The resin was rinsed with three column volumes of purified water in order to remove polar impurities. α-Mangostin was eluted by a wash of methanol. The eluent was monitored via HPLC-analysis. The fractions with high levels of α-mangostin were combined and evaporated to dryness. Crude α-mangostin (6.3 g) was obtained. The α-mangostin obtained was recrystallized in 100 mL 95% ethanol to obtain the final product. The α-mangostin was identified by GS-MS, NMR, IR and UV-VIS spectroscopy.

### The interaction between α-mangostin and serum proteins

Fluorescence emission spectra and synchronous spectra of α-mangostin and HSA or TRF solution: HSA or TRF (2.5 mL) was added to a 1 cm quartz cuvette, and then 1.0 mM α-mangostin (total volume = 100 μL) was added in potions with a microsyringe into the quartz cuvette for fluorescence titration. The α-mangostin concentrations in the cuvette were 0, 4, 8, 10, 16, 24, 32 or 40 μM. Slit widths of emission and excitation: 2.5 nm; scan rate: 240 nm/min; excitation wavelength: 282 nm. The emission spectra were obtained between 280 and 500 nm and the fluorescence intensities at the maximum emission wavelength were recorded.

In order to determine the synchronous fluorescence spectra and fluorescence polarization spectra of α-mangostin binding with HSA or TRF, the wavelength differences (Δλ) of fluorescence emission and excitation were set to 60 nm and 15 nm, respectively, and scan rate to 240 nm/min.

UV absorption spectra of α-mangostin and HSA or TRF solution were obtained by setting up 1 cm quartz cuvette with 8 different α-mangostin concentrations (0, 4, 8, 10, 16, 24, 32 or 40 μM) as described above using Tris-HCl buffer as UV blank correction. The ultraviolet absorption spectra were obtained between 200 and 500 nm.

### Molecular docking

Interaction models of drug and proteins were established by molecular simulation via DOCK 4.0 at SGI O2 workstation. The crystal structures of HSA and TRF were obtained from Brookhaven Protein Data Bank (encoding: 1H9Z and 1D4N, respectively). In the process of molecular docking, following some reports [46–50], we use the Chain A of HSA for the docking studies. Molecular structure of α-mangostin was built by ChemDraw and then molecular modeling based on the force field optimization of MM2 molecular mechanics was established. In the pretreatment process of molecular docking, receptor and ligand could be optimized with AutoDock Tools (ADT). Ligand had been handled with adding hydrogenation, adding charge and adding the atomic type, While the entire protein had been pretreatmented about deleting water, adding hydrogens and computing gasteiger by AutoDock Tools. The pretreatment of α-mangostin were automatically docked into the binding cavity of HSA/TRF by autodock 4.2. The active sites of HSA and TRF were confirmed through molecular docking and in order to find the best binding mode of ligand and receptor, we adopted the empirical potential-energy function as the evaluation function [51–53].

## Supporting Information

S1 Fig Schematic diagram of macroporous resin extraction apparatus. (TIFF)
