# Supplementary figures and images for: α-Mangostin Extraction from the Native Mangosteen (Garcinia mangostana L.) and the Binding Mechanisms of α-Mangostin to HSA or TRF
Source: PLoS One. 2016 Sep 1;11(9):e0161566. doi: 10.1371/journal.pone.0161566 (PMC5008840; doi:10.1371/journal.pone.0161566)

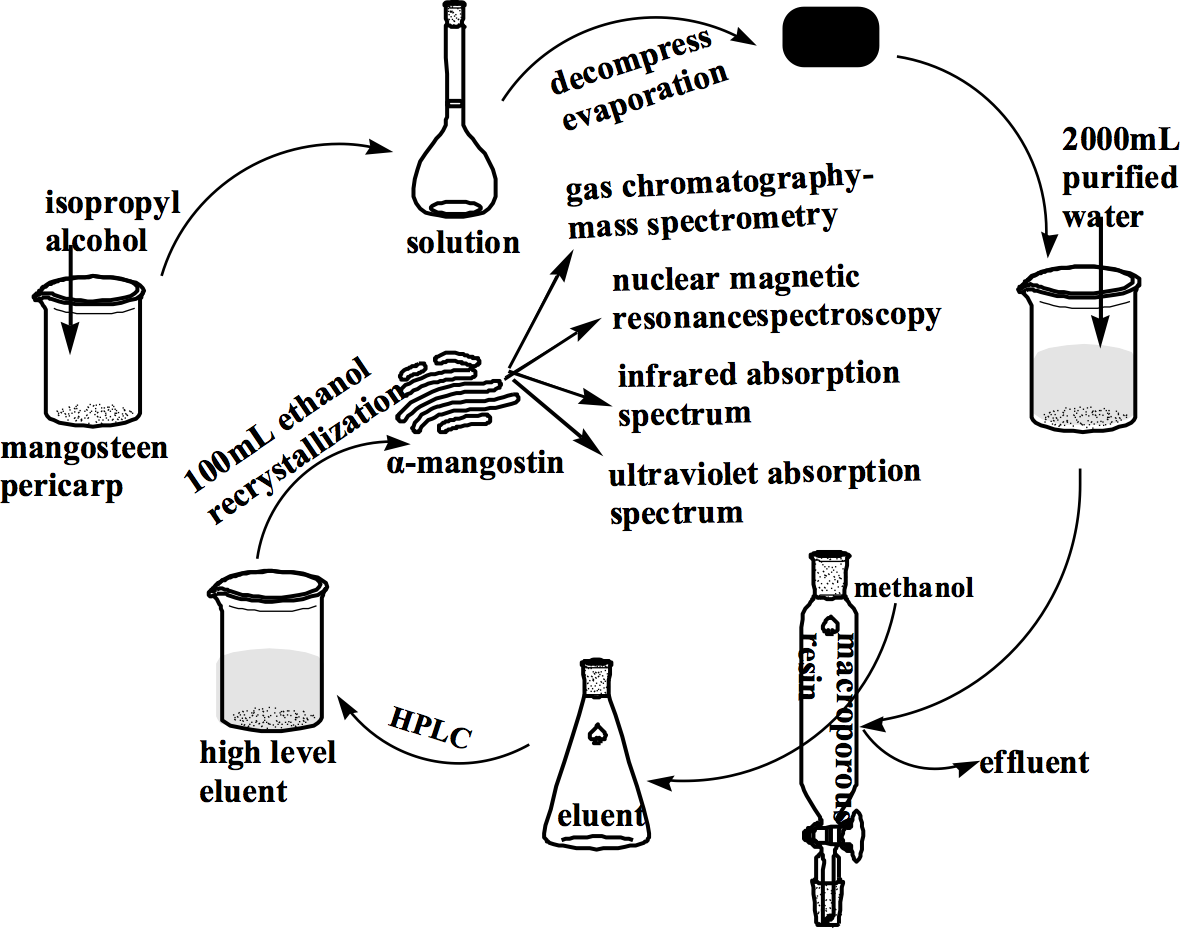

Supplement: S1 Fig — (TIFF) [file pone.0161566.s001.tiff]
